# Telomeric repeat-containing RNA TERRA: a noncoding RNA connecting telomere biology to genome integrity

**DOI:** 10.3389/fgene.2015.00143

**Published:** 2015-04-14

**Authors:** Emilio Cusanelli, Pascal Chartrand

**Affiliations:** ^1^Max F. Perutz Laboratories, Department of Chromosome Biology, University of ViennaVienna, Austria; ^2^Department of Biochemistry and Molecular Medicine, Université de MontréalMontréal, QC, Canada

**Keywords:** TERRA, telomere, R-loops, DNA damage response, telomerase, genome integrity, cancer

## Abstract

Telomeres are dynamic nucleoprotein structures that protect the ends of chromosomes from degradation and activation of DNA damage response. For this reason, telomeres are essential to genome integrity. Chromosome ends are enriched in heterochromatic marks and proper organization of telomeric chromatin is important to telomere stability. Despite their heterochromatic state, telomeres are transcribed giving rise to long noncoding RNAs (lncRNA) called TERRA (telomeric repeat-containing RNA). TERRA molecules play critical roles in telomere biology, including regulation of telomerase activity and heterochromatin formation at chromosome ends. Emerging evidence indicate that TERRA transcripts form DNA-RNA hybrids at chromosome ends which can promote homologous recombination among telomeres, delaying cellular senescence and sustaining genome instability. Intriguingly, TERRA RNA-telomeric DNA hybrids are involved in telomere length homeostasis of telomerase-negative cancer cells. Furthermore, TERRA transcripts play a role in the DNA damage response (DDR) triggered by dysfunctional telomeres. We discuss here recent developments on TERRA's role in telomere biology and genome integrity, and its implication in cancer.

## Introduction

Telomeres are nucleoprotein structures assembled at the extremities of eukaryotic chromosomes that protect chromosome ends. By doing so, telomeres prevent chromosome ends from being recognized as sites of DNA damage, protecting them from degradation and inappropriate recombination events due to erroneous activation of DNA repair pathways (Doksani and de Lange, 2014). Telomeres are also required for the complete replication of genomic DNA. Indeed, eukaryotic chromosomes are deemed to progressively shorten during cell divisions due to limitations of the semiconservative DNA replication machinery which is unable to fully replicate the extremities of linear DNA, a phenomenon known as the “end replication problem” (Hug and Lingner, 2006; Jain and Cooper, 2010). In the absence of maintenance mechanisms chromosome ends erode, telomeres become dysfunctional and are recognized as sites of DNA damage, leading to cellular senescence or rampant genome instability and apoptosis (Deng et al., 2008). In most eukaryotes, telomeres compensate for the end replication problem by recruiting the reverse transcriptase telomerase. Telomerase uses the 3′ end of chromosomes as primer to elongate chromosome ends by reverse transcription of the template region of its RNA moiety and in concerted action with the DNA replication machinery (Greider and Blackburn, 1985; Hug and Lingner, 2006). While telomerase activity is detected in some highly proliferative tissues, not sufficient amount of telomerase is expressed in human somatic cells which enter replicative senescence upon a defined number of cell divisions due to telomere erosion (Allsopp et al., 1992). Telomerase inactivation acts as a tumor suppressor mechanism as re-expression of this enzyme in human fibroblasts allows to bypass senescence, leading to cellular immortalization (Bodnar et al., 1998). Accordingly, 90% of human cancers reactivate telomerase activity, resulting in stabilized telomere length (Kim et al., 1994). For the reasons mentioned above, functional telomeres are essential to genome integrity and cell viability.

In order to exert their functions, telomeres rely on a highly conserved DNA structure which consists of a variable number of telomeric repeats (TTAGGG_n_ in mammalian cells) followed by a single-stranded G-rich 3′ overhang. Electron microscopy and super-resolution fluorescence imaging have shown that telomeric DNA forms higher order structures where the 3′ single-stranded overhang invades the homologous double-stranded region forming a telomeric loop (T-loop) (Griffith et al., 1999; Doksani et al., 2013). T-loop formation is believed to sequester the 3′ end of chromosomes, thereby preventing its recognition by the DNA damage machinery (de Lange, 2009; Doksani et al., 2013). Indeed, while essential component of telomeres, 3′ overhang is also a general feature of DNA double strand breaks (DSBs). Functional telomeres thus enable the cell to discriminate the natural chromosome ends from the harmful DSBs.

Telomeric DNA acts as docking site for telomere binding proteins which regulate telomere homeostasis and mediate telomere functions (de Lange, 2005). In mammalian cells, the protein complex shelterin is recruited at telomeres through the direct binding of its subunits TRF1 and TRF2 to the double-stranded telomeric DNA (Palm and de Lange, 2008). Telomere-bound TRF1 and TRF2 allow the recruitment of the other shelterin components TIN2, the TPP1/POT1 heterodimer, and Rap1 to chromosome ends (Sfeir and de Lange, 2012). At telomeres, shelterin proteins mediate distinct functions: TRF2 is required for T-loop formation and maintenance (Doksani et al., 2013), and for repression of ATM-mediated DNA damage response (DDR) as well as non-homologous end joining (NHEJ) (Karlseder et al., 2004; Denchi and de Lange, 2007; Palm and de Lange, 2008). TRF1 has a pivotal role in controlling replication of telomeric DNA (Sfeir et al., 2009; Zimmermann et al., 2014) while POT1 associates with TPP1 to bind the single-stranded 3′ overhang and repress ATR-mediated DDR by preventing the recruitment of replication protein A (RPA) (Denchi and de Lange, 2007). TIN2 is essential to the overall integrity of the shelterin complex as it links TPP1/POT1 heterodimer to TRF1 and TRF2, and stabilizes TRF1 and TRF2 association to telomeric DNA (Takai et al., 2011; Frescas and de Lange, 2014a,b). Rap1 interacts with TRF2 but its role in telomere biology is still unclear (Kabir et al., 2014).

A conserved feature of telomeres is their enrichment in heterochromatic marks. Human and mouse subtelomeres are heavily methylated through the activity of DNA methyltransferases DNMT1, DNMT3a, and DNMT3b (Gonzalo et al., 2006; Schoeftner and Blasco, 2010). Chromatin of mammalian telomeres is also under-acetylated and enriched in histone H3 tri-methylated at lysine 9 (H3K9me3) and H4K20me3 (Benetti et al., 2007). These posttranslational modifications are mediated by the histone methyltransferases (HMT) Suv39h and Suv4-20h. Loss of HMTs or DNMs results in over-elongated telomeres, indicating the important role of telomeric chromatin in telomere homeostasis and stability (Schoeftner and Blasco, 2010). Consistent with the highly conserved compacted state of chromosome ends, earlier studies have shown that reporter genes integrated in proximity to yeast, flies, and mammalian telomeres are transcriptionally silenced (Baur et al., 2001; Koering et al., 2002; Rusche et al., 2002; Mason et al., 2008), a phenomenon called “telomere position effect” or TPE.

In stark contrast with these findings and in defiance of the longstanding belief that chromosome ends are transcriptionally silenced, recent evidence has shown that telomeres are transcribed by RNA polymerase II, giving rise to a class of long noncoding RNAs containing telomeric repeats called TERRA (Azzalin et al., 2007; Schoeftner and Blasco, 2008). TERRA molecules have been detected in a variety of organisms, including yeast, zebrafish, mouse and human, and are believed to actively participate in the mechanisms regulating telomere homeostasis and telomere function (Luke et al., 2008; Schoeftner and Blasco, 2008). TERRA transcripts have been involved in the regulation of telomerase, formation of heterochromatin at telomeres and proper capping of chromosome ends. Nevertheless, the mechanisms of action of telomeric noncoding RNAs remain largely to be elucidated. In this review, we discuss recent evidence on the emerging role of TERRA acting at the interface between telomeric DNA and telomere binding proteins to regulate telomere biology and genome stability.

## Telomeric repeat-containing RNA TERRA in telomere biology

TERRA molecules are transcribed from the subtelomeric regions toward the chromosome ends and consist of subtelomeric-derived sequences and G-rich telomeric repeats (Azzalin et al., 2007; Schoeftner and Blasco, 2008). TERRA promoter regions have been identified at CpG islands present in a subset of human telomeres in proximity to their telomeric repeats tract. Consistently, DNA methylation at subtelomeric regions generally associates with decreased expression of TERRA (Yehezkel et al., 2008; Nergadze et al., 2009; Ng et al., 2009; Farnung et al., 2012). Very recently, a second class of TERRA promoters located 5–10 kilobases away from the telomeric repeats of 10 distinct human telomeres have been identified (Porro et al., 2014a). The presence of different types of promoters likely contributes to the length heterogeneity of TERRA transcripts. Several lines of evidence indicate that modifications of the heterochromatic state of chromosome ends regulate the expression of TERRA (Azzalin and Lingner, 2008; Schoeftner and Blasco, 2008; Caslini et al., 2009; Iglesias et al., 2011; Arnoult et al., 2012). The mechanisms regulating TERRA expression and TERRA biogenesis have been recently reviewed elsewhere and will not be covered in this review (Azzalin and Lingner, 2014; Cusanelli and Chartrand, 2014; Maicher et al., 2014). Interestingly, while TERRA transcription has been detected from all human and yeast telomeres analyzed so far, a recent study indicates that TERRA is mainly transcribed from only two telomeres in mouse (de Silanes et al., 2014). While it cannot be formerly excluded that telomeric RNAs containing only telomeric repeats are also expressed in mouse, this study reveals that TERRA transcribed from a single telomere can associate with multiple chromosome ends. This suggests that TERRA can act *in trans* in mammalian cells and further supports the view of TERRA as an essential player for the overall maintenance of telomeres and/or telomere function (de Silanes et al., 2014). In yeast, live cell imaging experiments have shown that TERRA molecules preferentially localize with their telomere of origin during S phase (Cusanelli et al., 2013). In this cellular context, it has been proposed that TERRA expression participates in telomerase-mediated re-lengthening of the TERRA transcribing telomere (see below) (Cusanelli et al., 2013). Less is known on the dynamics of TERRA localization in human cells where TERRA transcripts associate with only a subset of chromosome ends at a given time (Azzalin et al., 2007; Lai et al., 2013), while a fraction of telomeric RNAs also resides within the nucleoplasm (Porro et al., 2010), suggesting that TERRA molecules are not constitutively associated with telomeres.

**Figure 1:**
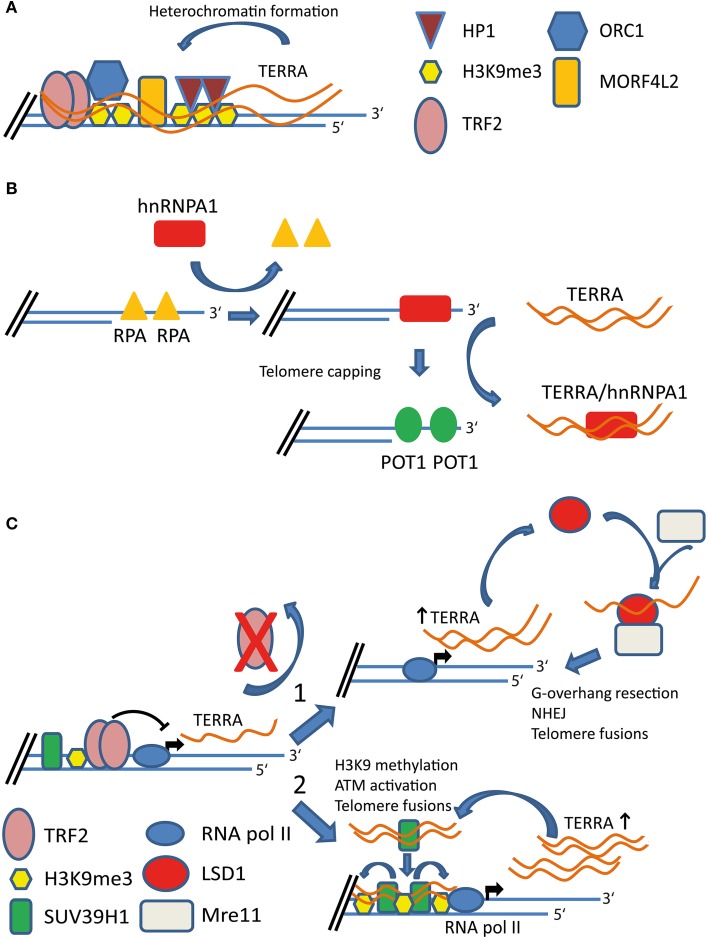
**Proposed functions of TERRA at functional and dysfunctional telomeres. (A)** TERRA expression promotes heterochromatin formation at telomeres. TERRA interacts with several proteins at telomeres including TRF2, H3K9me3, origin replication complex 1 (ORC1), HP1, and MORF4L2 proteins. TERRA molecules act as scaffold recruiting chromatin remodeling factors to chromosome ends. **(B)** Expression of TERRA is required for proper capping of telomeres. hnRNPA1 displaces RPA from telomeric single-stranded overhangs. TERRA transcripts interact with hnRNPA1. TERRA-hnRNPA1 interaction removes hnRNPA1 from chromosome ends allowing POT1 to bind the telomeric single-stranded overhangs. **(C)** TERRA participates to DNA damage response triggered by dysfunctional telomeres. Depletion of TRF2 results in dysfunctional telomeres and increased TERRA expression. TERRA interacts with lysine-specific demethylase 1 (LSD1). Elevated TERRA levels in TRF2-depleted cells promote nucleolytic processing of uncapped telomeres by favoring the recruitment of a LSD1-MRE11 complex at telomeres (1). TERRA molecules interact with SUV39H1 histone methyltransferase. TERRA-SUV39H1 interaction promotes H3K9 methylation (H3K9me3) at dysfunctional telomeres and chromosome end-to-end fusions (2).

How do TERRA transcripts associate with chromosome ends? Depletion of components of the nonsense mediated RNA decay (NMD) pathway or members of the heterogeneous nuclear ribonucleoprotein family (hnRNPs) which bind TERRA, increases localization of TERRA at chromosome ends without affecting its overall levels or stability (Azzalin et al., 2007; Lopez de Silanes et al., 2010). These findings suggest that TERRA molecules are actively displaced from telomeres and thus may be recruited at chromosome ends through interaction with stable constituents of the telomeric structure. In line with this view, it has been shown that TERRA associates with the shelterin components TRF1 and TRF2 (Deng et al., 2009b). This interaction is mediated by different TRF2 domains, including the amino-terminal GAR domain and carboxy-terminal myb domain (Deng et al., 2009b). In different studies, a number of other TERRA-binding proteins have been identified, including the heterochromatin protein 1 (HP1), SUV39H1, and MORF4L2, a component of the NuA2 histone acetyltransferase complex (Deng et al., 2009b; Lopez de Silanes et al., 2010; Scheibe et al., 2013; Porro et al., 2014a). Intriguingly, these proteins also localize at telomeres. TERRA transcripts have been proposed to promote or stabilize the recruitment of TERRA-binding proteins at chromosome ends (Deng et al., 2009b; Arnoult et al., 2012; Porro et al., 2014a). TERRA was also found to interact with tri-methylated histone H3K9me3 and depletion of TERRA molecules associates with a decrease in H3K9m3 and other heterochromatic marks at telomeres (Deng et al., 2009b). Altogether, this evidence has suggested that TERRA participates in heterochromatin formation at chromosome ends (Figure 1A) (Deng et al., 2009b; Arnoult et al., 2012). These findings support the emerging role of TERRA acting as a scaffold molecule to promote recruitment of proteins and enzymatic activities at telomeres.

## Connecting telomere biology and genome integrity

The interaction of TERRA with shelterin components is not the only mechanism through which TERRA molecules can associate with telomeres. Recent evidence has established that endogenous TERRA transcripts can base-pair with their template DNA strand, forming RNA:DNA hybrid structures known as R-loops (Balk et al., 2013; Pfeiffer et al., 2013; Arora et al., 2014; Yu et al., 2014). In R-loop structures, the RNA transcript anneals with the DNA template strand, displacing the complementary non-template strand which remains unpaired (Aguilera and Garcia-Muse, 2012). R-loops associated with G-rich sequences are involved in transcription termination (Skourti-Stathaki et al., 2011) and regulation of gene expression (Ginno et al., 2012). Recent findings have revealed the important role of R-loop structures during class switch recombination at the immunoglobulin heavy chains, where R-loops exceeding 1 kilobase in length are detected (Yu et al., 2003). While R-loops occur in natural context, they also pose a threat to genome integrity as their formation associates with mutations, recombination, replication stalling, and chromosome rearrangements (Aguilera and Garcia-Muse, 2012; Bermejo et al., 2012). For this reason, R-loop formation is tightly controlled within the cell and hazardous R-loop structures are removed by the activities of different enzymes such as RNase H (RNase H1 and 2) which degrades the RNA part of a DNA-RNA hybrid (Aguilera and Garcia-Muse, 2012); helicases, including Pif1 DNA helicase which is able to unwind DNA-RNA hybrid structures (Boule and Zakian, 2007; Paeschke et al., 2013); and the THO/TREX protein complex, initially identified for its involvement in transcription and mRNA export (Rondon et al., 2010); however, mutants of THO complex accumulate R-loop structures (Huertas and Aguilera, 2003).

**Figure 2:**
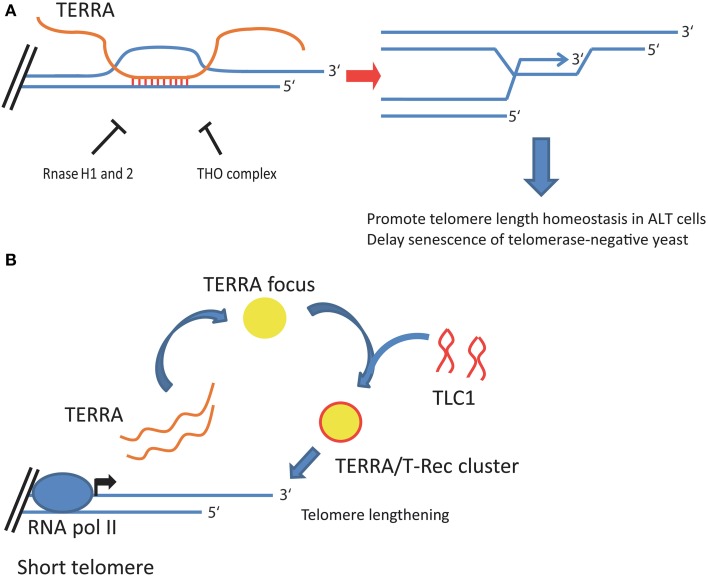
**Proposed roles of TERRA in telomere length homeostasis of telomerase-negative and telomerase-positive cells. (A)** In telomerase-negative cells, TERRA molecules form DNA:RNA hybrids, or R-loops, at telomeres. Telomeric R-loop formation is inhibited by RNase H1 and 2, and the THO complex in budding yeast, and is repressed by RNase H1 in ALT cancer cells. Telomeric R-loops promote homologous recombination among telomeres, which maintains telomere length homeostasis in ALT cancer cells and delays senescence of telomerase-negative yeast cells. **(B)** In telomerase-positive yeast cells, TERRA transcripts promote the formation of telomerase clusters at short telomeres in yeast. TERRA expression is induced when a telomere shortens and TERRA transcripts accumulate in a single focus localizing at the nuclear periphery. During S phase, a TERRA focus acts as a scaffold to bind and aggregate telomerase molecules (*TLC1*) into a TERRA/T-Rec cluster. TERRA/T-Rec cluster relocates to the short telomere expressing TERRA to mediate telomere elongation.

Recent evidence indicates that R-loops form at telomeres in yeast and in mammalian cells (Balk et al., 2013; Pfeiffer et al., 2013; Arora et al., 2014; Yu et al., 2014). In yeast, R-loops are detected at telomeres in WT cells but their formation is repressed by the endogenous RNase H1 and 2 enzymes and by the THO complex. Accordingly, telomeric R-loops accumulate in a RNase H1 and 2 double mutant strain (*rnh1 rnh201*) (Balk et al., 2013) as well as in THO complex mutant strains (*hpr1*, *thp2*, and *tho2*) (Pfeiffer et al., 2013; Yu et al., 2014) (Figure 2A). Interestingly, accumulation of telomeric R-loops promotes homologous recombination at telomeres. In particular, a recent study has shown that a telomerase-negative *rnh1 rnh201* yeast strain manifests a higher rate of telomere recombination than WT cells, which translates into a delayed onset of senescence in culture, expected to occur at 60–80 generations in telomerase-negative yeasts (Balk et al., 2013). The delay in senescence observed in telomerase-negative *rnh1 rnh201* strain is prevented by inactivation of *RAD52*, an essential regulator of homologous recombination (Wellinger and Zakian, 2012; Balk et al., 2013). Furthermore, overexpression of *RNH1* decreases telomere recombination and anticipates senescence of telomerase-negative cells (Balk et al., 2013). This evidence indicates that telomeric R-loops promote Rad52-mediated homologous recombination among telomeres (Lundblad and Blackburn, 1993; Wellinger and Zakian, 2012), delaying senescence of telomerase-negative cells (Balk et al., 2013). Similar results have been obtained in telomerase-negative THO mutant strains, where accumulation of TERRA at telomeric chromatin and consequent formation of telomeric R-loops promote recombination events among chromosome ends (Yu et al., 2014). Interestingly, in telomerase-positive cells, the THO complex also regulates chromosome end processing and prevents interference with telomeric DNA replication by regulating TERRA expression and its association at telomeres (Pfeiffer et al., 2013). These findings are consistent with a previous study showing that TERRA overexpression can promote processing of chromosome ends via the 5′–3′ exonuclease Exo1 (Pfeiffer and Lingner, 2012).

In a recent study, Arora and colleagues reported the presence of telomeric R-loops in telomerase-negative human cancer cells (Arora et al., 2014). The majority of human tumors reactivate telomerase activity to attain unlimited replicative capacity, but 10–15% of cancers maintain their telomeres in the absence of telomerase (Bryan et al., 1995). In these cells, telomere length homeostasis is achieved by homologous recombination-mediated mechanisms which, as in yeast, are known as alternative lengthening of telomeres or ALT (Bryan et al., 1995). While homologous recombination events among telomeres are an accepted common feature of ALT processes (Dunham et al., 2000; Conomos et al., 2013), the mechanisms triggering telomere recombination remain to be defined. Remarkably, Arora and colleagues have shown that ALT telomeres contain telomeric R-loop structures which play an essential role in telomere maintenance of ALT cells (Arora et al., 2014). The authors show that telomeric R-loops are tightly regulated by RNase H1, which localizes at telomeres in ALT cells but not in telomerase positive cells. Overexpression of RNase H1 decreases telomere recombination rate and leads to telomere shortening in ALT cells but not in telomerase-positive cells. In line with this evidence, TERRA transcripts localize within ALT-associated PML bodies (APBs) (Arora et al., 2014), distinct nuclear bodies of ALT cells where telomere recombination is believed to occur (Yeager et al., 1999). TERRA expression is induced in ALT cells (Ng et al., 2009; Arora et al., 2014) and recent evidence indicate that while TERRA levels decrease from S phase to G_2_ phase in telomerase-positive cancer cells (Porro et al., 2010), TERRA foci significantly increase during S phase and G_2_ in ALT cells (Flynn et al., 2015). Cell-cycle regulation of TERRA depends on the chromatin-remodeling protein ATRX, which loss or mutation correlate with ALT in human cancer (Heaphy et al., 2011; Lovejoy et al., 2012; Schwartzentruber et al., 2012). Indeed, depletion of ATRX in telomerase-positive cells also results in elevated TERRA levels and persistent TERRA foci in G_2_/M, suggesting that ATRX may act at telomeres by regulating TERRA expression and localization during cell cycle (Flynn et al., 2015). Altogether, these findings indicate that TERRA plays a major role in the maintenance of ALT telomeres. Telomeric R-loops thus represent novel players in telomere biology regulating telomere function and telomere stability of ALT cells.

## TERRA and DNA damage response at telomeres

TERRA transcripts have been proposed to participate in the proper telomere capping that prevents activation of DDR at chromosome ends. During telomeric DNA replication, exposed single-stranded DNA (ssDNA) is bound by the ssDNA binding protein RPA which is required for activation of the ATR checkpoint (Zou and Elledge, 2003; Verdun and Karlseder, 2006). The shelterin component POT1 acts in repressing ATR-mediated DDR at telomeres and it is believed to antagonize RPA for the binding to the telomeric ssDNA (Denchi and de Lange, 2007). *In vitro* evidence suggests that TERRA plays a role in the switch between RPA and POT1 at chromosome ends (Flynn et al., 2011). In particular, TERRA directly interacts with hnRNPA1 (Lopez de Silanes et al., 2010), which can displace RPA from telomeric ssDNA *in vitro* (Flynn et al., 2011). In this scenario, TERRA and hnRNPA1 cooperate to allow POT1 binding to telomeres at the expenses of RPA displacement after DNA replication (Figure 1B) (Flynn et al., 2011). It will be important to validate this mechanism *in vivo*.

In line with this view, emerging evidence indicates that altered TERRA expression or localization is involved in the activation of DDR at telomeres. Indeed, partial depletion of TERRA expressed from the single telomere 18 in mouse cells leads to DDR activation at different chromosome ends and widespread telomere dysfunction (de Silanes et al., 2014). Furthermore, partial down-regulation of TERRA expression (Deng et al., 2009b, 2012a) and unscheduled accumulation of TERRA transcripts at telomeres associate with activation of DDR at chromosome ends, with consequent formation of “telomere dysfunction-induced foci” (TIF) (Lopez de Silanes et al., 2010). Finally, depletion of TRF2, which activates the ATM kinase pathway at telomeres and results in telomere fusions through NHEJ (Takai et al., 2003; Denchi and de Lange, 2007), leads to up-regulation of TERRA (Caslini et al., 2009; Porro et al., 2014a,b). In this regard, the TRF2 homodimerization domain (TFRH) is required for repression of TERRA transcription (Porro et al., 2014a). Importantly, this domain is not involved in TERRA binding. The involvement of the TFRH domain in TERRA repression suggests that TRF2 may negatively regulate TERRA transcription through its activity on telomeric structure (Poulet et al., 2012; Porro et al., 2014a). Porro and colleagues recently studied the role of TERRA at dysfunctional telomeres in TRF2-deficient cells (Porro et al., 2014b). The authors show that TERRA up-regulation correlates with telomeric recruitment of the lysine-specific demethylase 1 (LSD1) (Porro et al., 2014b). LSD1 directly interacts with TERRA and MRE11 (Porro et al., 2014b), a subunit of the MRE11/RAD50/NBS1 (MRN) complex required for the resection of the telomeric 3′ overhang to promote chromosome fusion at dysfunctional telomeres (van Steensel et al., 1998; Deng et al., 2009a). Interestingly, TERRA expression stabilizes LSD1-MRE11 association *in vitro* and *in vivo* (Porro et al., 2014b). These findings suggest that increased expression of TERRA in TRF2-depleted cells may contribute to the activation of NHEJ by promoting MRE11 activity at uncapped telomeres (Figure 1C) (Porro et al., 2014b).

In addition to their implication in telomere end processing, a recent study indicates that TERRA transcripts can promote changes in chromatin structure of uncapped telomeres (Porro et al., 2014a). In particular, TERRA was shown to interact with the histone methyltransferase SUV39H1 and promote methylation of histone H3K9 upon TRF2 depletion by enhancing telomere association of SUV39H1. SUV39H1 is important for efficient end fusions of TRF2-depleted telomeres (Bartocci et al., 2014; Porro et al., 2014a). Furthermore, accumulation of H3K9me3 at uncapped telomeres may serve as a docking site for the recruitment of the Tip60/KAT5 acetyltransferase, which is required for ATM acetylation and activation (Murr et al., 2006; Porro et al., 2014a). Importantly, TERRA up-regulation upon TRF2 depletion occurs independently of the DDR and it seems to be an early event occurring in parallel or upstream of ATM activation (Porro et al., 2014a). These findings indicate that TERRA can actively participate in the DDR triggered by dysfunctional telomeres by promoting the association of telomere end processing and chromatin remodeling factors at telomeres (Figure 1C).

## TERRA and telomerase

Ever since the discovery of TERRA, it was presumed that TERRA transcripts can regulate telomerase activity (Schoeftner and Blasco, 2008). The 3′ end of TERRA is indeed complementary to the template region of telomerase RNA and telomerase associates with TERRA transcripts *in vivo*, although it is not known if TERRA binds the template region of hTR (Redon et al., 2010). TERRA-mimicking oligonucleotides inhibit telomerase activity *in vitro* (Schoeftner and Blasco, 2008; Redon et al., 2010). Nevertheless, a role for TERRA as negative regulator of telomerase *in vivo* is still unclear, since overexpression of TERRA has no effect on telomerase activity in human cancer cells (Farnung et al., 2012). In yeast, TERRA expression is induced by telomere shortening and TERRA molecules organize telomerase activity at their telomere of origin by acting as a scaffold to promote telomerase nucleation and formation of telomerase recruitment clusters (or T-Recs) (Figure 2B) (Gallardo et al., 2011; Cusanelli et al., 2013). Accordingly, TERRA interacts with yeast telomerase RNA *TLC1 in vivo* and TERRA/T-Recs complexes preferentially localize to TERRA-expressing short telomeres (Cusanelli et al., 2013). Further studies *in vivo* will be required to understand the role of TERRA in the regulation of telomerase in mammalian cells.

## Concluding remarks

It has become increasingly clear that TERRA transcripts actively participate in the various functions of telomeres and in telomere stability. Their expression and localization must be kept in check as TERRA can contribute by different means to the DDR triggered at dysfunctional telomeres, which poses a threat to genome integrity. Beside a role for TERRA in tumor cells, telomere dysfunction also occurs during replicative senescence (d'Adda di Fagagna et al., 2003), suggesting that TERRA may play a role in aging and age-related diseases. Dysfunction in TERRA expression is linked to diseases, like the immunodeficiency, centromere instability, and facial anomalies (ICF) syndrome, a rare autosomal recessive immune disorder caused by mutations in the DNA methyltransferase gene DNMT3b (Xu et al., 1999). TERRA is overexpressed in primary cells of ICF patients, possibly due to the hypomethylated state of their subtelomeric promoters (Yehezkel et al., 2008; Deng et al., 2010). Other diseases, like telomeropathies, may be associated with TERRA mis-regulation.

Major questions are still unanswered in the field and several challenges lay ahead. First and foremost, a well-established system to efficiently deplete total TERRA in cells remains to be developed. Current approaches, using RNAi or antisense oligonucleotides, only partially deplete TERRA levels. As a role for TERRA as a scaffold molecule involved in the recruitment and organization of enzymatic activities at telomeres is emerging, a major challenge will be to determine how these various activities are organized by TERRA according to the state of a telomere (i.e., capped vs. uncapped, short vs. long).

Telomerase is an established key target for cancer therapies (Harley, 2008). Yet recent evidence indicates that telomerase-positive tumor cells can develop resistance to telomerase-targeting cancer therapies by engaging ALT-mediated mechanisms (Chen et al., 2010; Hu et al., 2012). TERRA is induced in human and mouse tumors (Deng et al., 2012b) and may act as regulator of telomerase (Cusanelli et al., 2013). TERRA also plays a major role in telomere length homeostasis in ALT cancer cells through formation of telomeric R-loops (Arora et al., 2014). Targeting TERRA in tumor cells may impair telomerase activity while also preventing the development of ALT-mediated resistance mechanisms, making TERRA an attractive therapeutic target.

### Conflict of interest statement

The authors declare that the research was conducted in the absence of any commercial or financial relationships that could be construed as a potential conflict of interest.

## References

[B1] Aguilera A. Garcia-Muse T. (2012). R loops: from transcription byproducts to threats to genome stability. Mol. Cell 46, 115–124. 10.1016/j.molcel.2012.04.009 22541554

[B2] Allsopp R. C. Vaziri H. Patterson C. Goldstein S. Younglai E. V. Futcher A. B.. (1992). Telomere length predicts replicative capacity of human fibroblasts. Proc. Natl. Acad. Sci. U.S.A. 89, 10114–10118. 10.1073/pnas.89.21.10114 1438199 PMC50288

[B3] Arnoult N. Van Beneden A. Decottignies A. (2012). Telomere length regulates TERRA levels through increased trimethylation of telomeric H3K9 and HP1alpha. Nat. Struct. Mol. Biol. 19, 948–956. 10.1038/nsmb.2364 22922742

[B4] Arora R. Lee Y. Wischnewski H. Brun C. M. Schwarz T. Azzalin C. M. (2014). RNaseH1 regulates TERRA-telomeric DNA hybrids and telomere maintenance in ALT tumour cells. Nat. Commun. 5, 5220. 10.1038/ncomms6220 25330849 PMC4218956

[B5] Azzalin C. M. Lingner J. (2008). Telomeres: the silence is broken. Cell Cycle 7, 1161–1165. 10.4161/cc.7.9.5836 18418035

[B6] Azzalin C. M. Lingner J. (2014). Telomere functions grounding on TERRA firma. Trends Cell Biol. 1, 29–36. 10.1016/j.tcb.2014.08.007 25257515

[B7] Azzalin C. M. Reichenbach P. Khoriauli L. Giulotto E. Lingner J. (2007). Telomeric repeat containing RNA and RNA surveillance factors at mammalian chromosome ends. Science 318, 798–801. 10.1126/science.1147182 17916692

[B8] Balk B. Maicher A. Dees M. Klermund J. Luke-Glaser S. Bender K.. (2013). Telomeric RNA-DNA hybrids affect telomere-length dynamics and senescence. Nat. Struct. Mol. Biol. 20, 1199–1205. 10.1038/nsmb.2662 24013207

[B9] Bartocci C. Diedrich J. K. Ouzounov I. Li J. Piunti A. Pasini D.. (2014). Isolation of chromatin from dysfunctional telomeres reveals an important role for Ring1b in NHEJ-mediated chromosome fusions. Cell Rep. 7, 1320–1332. 10.1016/j.celrep.2014.04.002 24813883 PMC4054697

[B10] Baur J. A. Zou Y. Shay J. W. Wright W. E. (2001). Telomere position effect in human cells. Science 292, 2075–2077. 10.1126/science.1062329 11408657

[B11] Benetti R. Garcia-Cao M. Blasco M. A. (2007). Telomere length regulates the epigenetic status of mammalian telomeres and subtelomeres. Nat. Genet. 39, 243–250. 10.1038/ng1952 17237781

[B12] Bermejo R. Lai M. S. Foiani M. (2012). Preventing replication stress to maintain genome stability: resolving conflicts between replication and transcription. Mol. Cell 45, 710–718. 10.1016/j.molcel.2012.03.001 22464441

[B13] Bodnar A. G. Ouellette M. Frolkis M. Holt S. E. Chiu C. P. Morin G. B.. (1998). Extension of life-span by introduction of telomerase into normal human cells. Science 279, 349–352. 10.1126/science.279.5349.349 9454332

[B14] Boule J. B. Zakian V. A. (2007). The yeast Pif1p DNA helicase preferentially unwinds RNA DNA substrates. Nucleic Acids Res. 35, 5809–5818. 10.1093/nar/gkm613 17720711 PMC2034482

[B15] Bryan T. M. Englezou A. Gupta J. Bacchetti S. Reddel R. R. (1995). Telomere elongation in immortal human-cells without detectable telomerase activity. EMBO J. 14, 4240–4248. 7556065 10.1002/j.1460-2075.1995.tb00098.x PMC394507

[B16] Caslini C. Connelly J. A. Serna A. Broccoli D. Hess J. L. (2009). MLL associates with telomeres and regulates telomeric repeat-containing RNA transcription. Mol. Cell. Biol. 29, 4519–4526. 10.1128/MCB.00195-09 19528237 PMC2725733

[B17] Chen W. Xiao B. K. Liu J. P. Chen S. M. Tao Z. Z. (2010). Alternative lengthening of telomeres in hTERT-inhibited laryngeal cancer cells. Cancer Sci. 101, 1769–1776. 10.1111/j.1349-7006.2010.01611.x 20545697 PMC11159073

[B18] Conomos D. Pickett H. A. Reddel R. R. (2013). Alternative lengthening of telomeres: remodeling the telomere architecture. Front. Oncol. 3:27. 10.3389/fonc.2013.00027 23429284 PMC3576624

[B19] Cusanelli E. Chartrand P. (2014). Telomeric noncoding RNA: telomeric repeat-containing RNA in telomere biology. Wiley Interdiscip. Rev. RNA 5, 407–419. 10.1002/wrna.1220 24523222

[B20] Cusanelli E. Romero C. A. Chartrand P. (2013). Telomeric noncoding RNA TERRA is induced by telomere shortening to nucleate telomerase molecules at short telomeres. Mol. Cell 51, 780–791. 10.1016/j.molcel.2013.08.029 24074956

[B21] d'Adda di Fagagna F. Reaper P. M. Clay-Farrace L. Fiegler H. Carr P. Von Zglinicki T.. (2003). A DNA damage checkpoint response in telomere-initiated senescence. Nature 426, 194–198. 10.1038/nature02118 14608368

[B22] de Lange T. (2005). Shelterin: the protein complex that shapes and safeguards human telomeres. Genes Dev. 19, 2100–2110. 10.1101/gad.1346005 16166375

[B23] de Lange T. (2009). How telomeres solve the end-protection problem. Science 326, 948–952. 10.1126/science.1170633 19965504 PMC2819049

[B24] Denchi E. L. de Lange T. (2007). Protection of telomeres through independent control of ATM and ATR by TRF2 and POT1. Nature 448, 1068–1071. 10.1038/nature06065 17687332

[B25] Deng Y. Chan S. S. Chang S. (2008). Telomere dysfunction and tumour suppression: the senescence connection. Nat. Rev. Cancer 8, 450–458. 10.1038/nrc2393 18500246 PMC3688269

[B26] Deng Y. Guo X. Ferguson D. O. Chang S. (2009a). Multiple roles for MRE11 at uncapped telomeres. Nature 460, 914–918. 10.1038/nature08196 19633651 PMC2760383

[B27] Deng Z. Campbell A. E. Lieberman P. M. (2010). TERRA, CpG methylation and telomere heterochromatin: lessons from ICF syndrome cells. Cell Cycle 9, 69–74. 10.4161/cc.9.1.10358 20016274 PMC3664275

[B28] Deng Z. Norseen J. Wiedmer A. Riethman H. Lieberman P. M. (2009b). TERRA RNA binding to TRF2 facilitates heterochromatin formation and ORC recruitment at telomeres. Mol. Cell 35, 403–413. 10.1016/j.molcel.2009.06.025 19716786 PMC2749977

[B29] Deng Z. Wang Z. Stong N. Plasschaert R. Moczan A. Chen H. S.. (2012a). A role for CTCF and cohesin in subtelomere chromatin organization, TERRA transcription, and telomere end protection. EMBO J. 31, 4165–4178. 10.1038/emboj.2012.266 23010778 PMC3492729

[B30] Deng Z. Wang Z. Xiang C. Molczan A. Baubet V. Conejo-Garcia J. (2012b). Formation of telomeric repeat-containing RNA (TERRA) foci in highly proliferating mouse cerebellar neuronal progenitors and medulloblastoma. J. Cell Sci. 125, 4383–4394 10.1242/jcs.108118 22641694 PMC3516443

[B31] de Silanes I. L. Grana O. De Bonis M. L. Dominguez O. Pisano D. G. Blasco M. A. (2014). Identification of TERRA locus unveils a telomere protection role through association to nearly all chromosomes. Nat. Commun. 5, 4723. 10.1038/ncomms5723 25182072 PMC4164772

[B32] Doksani Y. de Lange T. (2014). The role of double-strand break repair pathways at functional and dysfunctional telomeres. Cold Spring Harb. Perspect. Biol. 6, 345–356. 10.1101/cshperspect.a016576 25228584 PMC4292156

[B33] Doksani Y. Wu J. Y. de Lange T. Zhuang X. (2013). Super-resolution fluorescence imaging of telomeres reveals TRF2-dependent T-loop formation. Cell 155, 345–356. 10.1016/j.cell.2013.09.048 24120135 PMC4062873

[B34] Dunham M. A. Neumann A. A. Fasching C. L. Reddel R. R. (2000). Telomere maintenance by recombination in human cells. Nat. Genet. 26, 447–450. 10.1038/82586 11101843

[B35] Farnung B. O. Brun C. M. Arora R. Lorenzi L. E. Azzalin C. M. (2012). Telomerase efficiently elongates highly transcribing telomeres in human cancer cells. PLoS ONE 7:e35714. 10.1371/journal.pone.0035714 22558207 PMC3338753

[B36] Flynn R. L. Centore R. C. O'Sullivan R. J. Rai R. Tse A. Songyang Z.. (2011). TERRA and hnRNPA1 orchestrate an RPA-to-POT1 switch on telomeric single-stranded DNA. Nature 471, 532–536. 10.1038/nature09772 21399625 PMC3078637

[B37] Flynn R. L. Cox K. E. Jeitany M. Wakimoto H. Bryll A. R. Ganem N. J.. (2015). Alternative lengthening of telomeres renders cancer cells hypersensitive to ATR inhibitors. Science 347, 273–277. 10.1126/science.1257216 25593184 PMC4358324

[B38] Frescas D. de Lange T. (2014a). TRF2-tethered TIN2 can mediate telomere protection by TPP1/POT1. Mol. Cell. Biol. 34, 1349–1362. 10.1128/MCB.01052-13 24469404 PMC3993560

[B39] Frescas D. de Lange T. (2014b). Binding of TPP1 protein to TIN2 protein is required for POT1a,b protein-mediated telomere protection. J. Biol. Chem. 289, 24180–24187. 10.1074/jbc.M114.592592 25056954 PMC4148849

[B40] Gallardo F. Laterreur N. Cusanelli E. Ouenzar F. Querido E. Wellinger R. J.. (2011). Live cell imaging of telomerase RNA dynamics reveals cell cycle-dependent clustering of telomerase at elongating telomeres. Mol. Cell 44, 819–827. 10.1016/j.molcel.2011.09.020 22152484

[B41] Ginno P. A. Lott P. L. Christensen H. C. Korf I. Chedin F. (2012). R-loop formation is a distinctive characteristic of unmethylated human CpG island promoters. Mol. Cell 45, 814–825. 10.1016/j.molcel.2012.01.017 22387027 PMC3319272

[B42] Gonzalo S. Jaco I. Fraga M. F. Chen T. Li E. Esteller M.. (2006). DNA methyltransferases control telomere length and telomere recombination in mammalian cells. Nat. Cell Biol. 8, 416–424. 10.1038/ncb1386 16565708

[B43] Greider C. W. Blackburn E. H. (1985). Identification of a specific telomere terminal transferase activity in Tetrahymena extracts. Cell 43, 405–413. 10.1016/0092-8674(85)90170-9 3907856

[B44] Griffith J. D. Comeau L. Rosenfield S. Stansel R. M. Bianchi A. Moss H.. (1999). Mammalian telomeres end in a large duplex loop. Cell 97, 503–514. 10.1016/S0092-8674(00)80760-6 10338214

[B45] Harley C. B. (2008). Telomerase and cancer therapeutics. Nat. Rev. Cancer 8, 167–179. 10.1038/nrc2275 18256617

[B46] Heaphy C. M. de Wilde R. F. Jiao Y. Klein A. P. Edil B. H. Shi C.. (2011). Altered telomeres in tumors with ATRX and DAXX mutations. Science 333, 425. 10.1126/science.1207313 21719641 PMC3174141

[B47] Hu J. Hwang S. S. Liesa M. Gan B. Sahin E. Jaskelioff M.. (2012). Antitelomerase therapy provokes ALT and mitochondrial adaptive mechanisms in cancer. Cell 148, 651–663. 10.1016/j.cell.2011.12.028 22341440 PMC3286017

[B48] Huertas P. Aguilera A. (2003). Cotranscriptionally formed DNA:RNA hybrids mediate transcription elongation impairment and transcription-associated recombination. Mol. Cell 12, 711–721. 10.1016/j.molcel.2003.08.010 14527416

[B49] Hug N. Lingner J. (2006). Telomere length homeostasis. Chromosoma 115, 413–425. 10.1007/s00412-006-0067-3 16741708

[B50] Iglesias N. Redon S. Pfeiffer V. Dees M. Lingner J. Luke B. (2011). Subtelomeric repetitive elements determine TERRA regulation by Rap1/Rif and Rap1/Sir complexes in yeast. EMBO Rep. 12, 587–593. 10.1038/embor.2011.73 21525956 PMC3128280

[B51] Jain D. Cooper J. P. (2010). Telomeric strategies: means to an end. Annu. Rev. Genet. 44, 243–269. 10.1146/annurev-genet-102108-134841 21047259

[B52] Kabir S. Hockemeyer D. de Lange T. (2014). TALEN gene knockouts reveal no requirement for the conserved human shelterin protein Rap1 in telomere protection and length regulation. Cell Rep. 9, 1273–1280. 10.1016/j.celrep.2014.10.014 25453752 PMC4254571

[B53] Karlseder J. Hoke K. Mirzoeva O. K. Bakkenist C. Kastan M. B. Petrini J. H.. (2004). The telomeric protein TRF2 binds the ATM kinase and can inhibit the ATM-dependent DNA damage response. PLoS Biol. 2:E240. 10.1371/journal.pbio.0020240 15314656 PMC509302

[B54] Kim N. W. Piatyszek M. A. Prowse K. R. Harley C. B. West M. D. Ho P. L.. (1994). Specific association of human telomerase activity with immortal cells and cancer. Science 266, 2011–2015. 10.1126/science.7605428 7605428

[B55] Koering C. E. Pollice A. Zibella M. P. Bauwens S. Puisieux A. Brunori M.. (2002). Human telomeric position effect is determined by chromosomal context and telomeric chromatin integrity. EMBO Rep. 3, 1055–1061. 10.1093/embo-reports/kvf215 12393752 PMC1307600

[B56] Lai L. T. Lee P. J. Zhang L. F. (2013). Immunofluorescence protects RNA signals in simultaneous RNA-DNA FISH. Exp. Cell Res. 319, 46–55. 10.1016/j.yexcr.2012.11.009 23164508

[B57] Lopez de Silanes I. Stagno d'Alcontres M. Blasco M. A. (2010). TERRA transcripts are bound by a complex array of RNA-binding proteins. Nat. Commun. 1, 33. 10.1038/ncomms1032 20975687

[B58] Lovejoy C. A. Li W. Reisenweber S. Thongthip S. Bruno J. de Lange T. (2012). Loss of ATRX, genome instability, and an altered DNA damage response are hallmarks of the alternative lengthening of telomeres pathway. PLoS Genet. 8:e1002772 10.1371/journal.pgen.1002772 22829774 PMC3400581

[B59] Luke B. Panza A. Redon S. Iglesias N. Li Z. Lingner J. (2008). The Rat1p 5′ to 3′ exonuclease degrades telomeric repeat-containing RNA and promotes telomere elongation in *Saccharomyces cerevisiae*. Mol. Cell 32, 465–477. 10.1016/j.molcel.2008.10.019 19026778

[B60] Lundblad V. Blackburn E. H. (1993). An alternative pathway for yeast telomere maintenance rescues est1- senescence. Cell 73, 347–360. 10.1016/0092-8674(93)90234-H 8477448

[B61] Maicher A. Lockhart A. Luke B. (2014). Breaking new ground: digging into TERRA function. Biochim. Biophys. Acta 1839, 387–394. 10.1016/j.bbagrm.2014.03.012 24698720

[B62] Mason J. M. Frydrychova R. C. Biessmann H. (2008). Drosophila telomeres: an exception providing new insights. Bioessays 30, 25–37. 10.1091/mbc.E02-03-0175 18081009 PMC2804870

[B63] Murr R. Loizou J. I. Yang Y. G. Cuenin C. Li H. Wang Z. Q.. (2006). Histone acetylation by Trrap-Tip60 modulates loading of repair proteins and repair of DNA double-strand breaks. Nat. Cell Biol. 8, 91–99. 10.1038/ncb1343 16341205

[B64] Nergadze S. G. Farnung B. O. Wischnewski H. Khoriauli L. Vitelli V. Chawla R.. (2009). CpG-island promoters drive transcription of human telomeres. RNA 15, 2186–2194. 10.1261/rna.1748309 19850908 PMC2779677

[B65] Ng L. J. Cropley J. E. Pickett H. A. Reddel R. R. Suter C. M. (2009). Telomerase activity is associated with an increase in DNA methylation at the proximal subtelomere and a reduction in telomeric transcription. Nucleic Acids Res. 37, 1152–1159. 10.1093/nar/gkn1030 19129228 PMC2651807

[B66] Paeschke K. Bochman M. L. Garcia P. D. Cejka P. Frieman K. L. Kowalczykowski S. C.. (2013). Pif1 family helicases suppress genome instability at G-quadruplex motifs. Nature 497, 458–462. 10.1038/nature12149 23657261 PMC3680789

[B67] Palm W. de Lange T. (2008). How shelterin protects mammalian telomeres. Annu. Rev. Genet. 42, 301–334. 10.1146/annurev.genet.41.110306.130350 18680434

[B68] Pfeiffer V. Crittin J. Grolimund L. Lingner J. (2013). The THO complex component Thp2 counteracts telomeric R-loops and telomere shortening. EMBO J. 32, 2861–2871. 10.1038/emboj.2013.217 24084588 PMC3817467

[B69] Pfeiffer V. Lingner J. (2012). TERRA promotes telomere shortening through exonuclease 1-mediated resection of chromosome ends. PLoS Genet. 8:e1002747. 10.1371/journal.pgen.1002747 22719262 PMC3375253

[B70] Porro A. Feuerhahn S. Delafontaine J. Riethman H. Rougemont J. Lingner J. (2014a). Functional characterization of the TERRA transcriptome at damaged telomeres. Nat. Commun. 5, 5379. 10.1038/ncomms6379 25359189 PMC4264578

[B71] Porro A. Feuerhahn S. Lingner J. (2014b). TERRA-reinforced association of LSD1 with MRE11 promotes processing of uncapped telomeres. Cell Rep. 6, 765–776. 10.1016/j.celrep.2014.01.022 24529708

[B72] Porro A. Feuerhahn S. Reichenbach P. Lingner J. (2010). Molecular dissection of telomeric repeat-containing RNA biogenesis unveils the presence of distinct and multiple regulatory pathways. Mol. Cell. Biol. 30, 4808–4817. 10.1128/MCB.00460-10 20713443 PMC2950545

[B73] Poulet A. Pisano S. Faivre-Moskalenko C. Pei B. Tauran Y. Haftek-Terreau Z.. (2012). The N-terminal domains of TRF1 and TRF2 regulate their ability to condense telomeric DNA. Nucleic Acids Res. 40, 2566–2576. 10.1093/nar/gkr1116 22139926 PMC3315331

[B74] Redon S. Reichenbach P. Lingner J. (2010). The non-coding RNA TERRA is a natural ligand and direct inhibitor of human telomerase. Nucleic Acids Res. 38, 5797–5806. 10.1093/nar/gkq296 20460456 PMC2943627

[B75] Rondon A. G. Jimeno S. Aguilera A. (2010). The interface between transcription and mRNP export: from THO to THSC/TREX-2. Biochim. Biophys. Acta 1799, 533–538. 10.1016/j.bbagrm.2010.06.002 20601280

[B76] Rusche L. N. Kirchmaier A. L. Rine J. (2002). Ordered nucleation and spreading of silenced chromatin in *Saccharomyces cerevisiae*. Mol. Biol. Cell 13, 2207–2222. 10.1091/mbc.E02-03-0175 12134062 PMC117306

[B77] Scheibe M. Arnoult N. Kappei D. Buchholz F. Decottignies A. Butter F.. (2013). Quantitative interaction screen of telomeric repeat-containing RNA reveals novel TERRA regulators. Genome Res. 23, 2149–2157. 10.1101/gr.151878.112 23921659 PMC3847783

[B78] Schoeftner S. Blasco M. A. (2008). Developmentally regulated transcription of mammalian telomeres by DNA-dependent RNA polymerase II. Nat. Cell Biol. 10, 228–236. 10.1038/ncb1685 18157120

[B79] Schoeftner S. Blasco M. A. (2010). Chromatin regulation and non-coding RNAs at mammalian telomeres. Semin. Cell Dev. Biol. 21, 186–193. 10.1016/j.semcdb.2009.09.015 19815087

[B80] Schwartzentruber J. Korshunov A. Liu X. Y. Jones D. T. Pfaff E. Jacob K.. (2012). Driver mutations in histone H3.3 and chromatin remodelling genes in paediatric glioblastoma. Nature 482, 226–231. 10.1038/nature10833 22286061

[B81] Sfeir A. de Lange T. (2012). Removal of shelterin reveals the telomere end-protection problem. Science 336, 593–597. 10.1126/science.1218498 22556254 PMC3477646

[B82] Sfeir A. Kosiyatrakul S. T. Hockemeyer D. MacRae S. L. Karlseder J. Schildkraut C. L.. (2009). Mammalian telomeres resemble fragile sites and require TRF1 for efficient replication. Cell 138, 90–103. 10.1016/S9999-9994(09)20370-9 19596237 PMC2723738

[B83] Skourti-Stathaki K. Proudfoot N. J. Gromak N. (2011). Human senataxin resolves RNA/DNA hybrids formed at transcriptional pause sites to promote Xrn2-dependent termination. Mol. Cell 42, 794–805. 10.1016/j.molcel.2011.04.026 21700224 PMC3145960

[B84] Takai H. Smogorzewska A. de Lange T. (2003). DNA damage foci at dysfunctional telomeres. Curr. Biol. 13, 1549–1556. 10.1016/S0960-9822(03)00542-6 12956959

[B85] Takai K. K. Kibe T. Donigian J. R. Frescas D. de Lange T. (2011). Telomere protection by TPP1/POT1 requires tethering to TIN2. Mol. Cell 44, 647–659. 10.1016/j.molcel.2011.08.043 22099311 PMC3222871

[B86] van Steensel B. Smogorzewska A. de Lange T. (1998). TRF2 protects human telomeres from end-to-end fusions. Cell 92, 401–413. 10.1016/S0092-8674(00)80932-0 9476899

[B87] Verdun R. E. Karlseder J. (2006). The DNA damage machinery and homologous recombination pathway act consecutively to protect human telomeres. Cell 127, 709–720. 10.1016/j.cell.2006.09.034 17110331

[B88] Wellinger R. J. Zakian V. A. (2012). Everything you ever wanted to know about *Saccharomyces cerevisiae* telomeres: beginning to end. Genetics 191, 1073–1105. 10.1534/genetics.111.137851 22879408 PMC3415994

[B89] Xu G. L. Bestor T. H. Bourc'his D. Hsieh C. L. Tommerup N. Bugge M.. (1999). Chromosome instability and immunodeficiency syndrome caused by mutations in a DNA methyltransferase gene. Nature 402, 187–191. 10.1038/46214 10647011

[B90] Yeager T. R. Neumann A. A. Englezou A. Huschtscha L. I. Noble J. R. Reddel R. R. (1999). Telomerase-negative immortalized human cells contain a novel type of promyelocytic leukemia (PML) body. Cancer Res. 59, 4175–4179. 10485449

[B91] Yehezkel S. Segev Y. Viegas-Pequignot E. Skorecki K. Selig S. (2008). Hypomethylation of subtelomeric regions in ICF syndrome is associated with abnormally short telomeres and enhanced transcription from telomeric regions. Hum. Mol. Genet. 17, 2776–2789. 10.1093/hmg/ddn177 18558631

[B92] Yu K. Chedin F. Hsieh C. L. Wilson T. E. Lieber M. R. (2003). R-loops at immunoglobulin class switch regions in the chromosomes of stimulated B cells. Nat. Immunol. 4, 442–451. 10.1038/ni919 12679812

[B93] Yu T. Y. Kao Y. W. Lin J. J. (2014). Telomeric transcripts stimulate telomere recombination to suppress senescence in cells lacking telomerase. Proc. Natl. Acad. Sci. U.S.A. 111, 3377–3382. 10.1073/pnas.1307415111 24550456 PMC3948247

[B94] Zimmermann M. Kibe T. Kabir S. de Lange T. (2014). TRF1 negotiates TTAGGG repeat-associated replication problems by recruiting the BLM helicase and the TPP1/POT1 repressor of ATR signaling. Genes Dev. 28, 2477–2491. 10.1101/gad.251611.114 25344324 PMC4233241

[B95] Zou L. Elledge S. J. (2003). Sensing DNA damage through ATRIP recognition of RPA-ssDNA complexes. Science 300, 1542–1548. 10.1126/science.1083430 12791985

